# Plant-Based No Added Fat or American Heart Association Diets, Impact on Cardiovascular Risk in Obese Hypercholesterolemic Children and Their Parents

**DOI:** 10.1016/j.jpeds.2014.12.058

**Published:** 2015-02-12

**Authors:** Michael Macknin, Tammie Kong, Adam Weier, Sarah Worley, Anne S. Tang, Naim Alkhouri, Mladen Golubic

**Affiliations:** Department of General Pediatrics, Cleveland Clinic Children’s; Department of Nutrition, Case Western Reserve University; Department of Nutrition, Case Western Reserve University; currently Nutrition and Food Service, VA Eastern Colorado Healthcare System; Department of Quantitative Health Sciences, Cleveland Clinic; Department of Quantitative Health Sciences, Cleveland Clinic; Department of Pediatric Gastroenterology, Cleveland Clinic Children’s; Center for Lifestyle Medicine, Cleveland Clinic

## Abstract

**Objective:**

To perform a randomized trial to determine if there is cardiovascular disease (CVD) risk reduction from a plant-based no added fat diet (PB) and the American Heart Association Diet (AHA) in children.

**Study design:**

Four-week (4/20/2013-5/18/2013) prospective randomized trial in a large Midwestern hospital system’s predominantly middle class outpatient pediatric practices. Thirty children (9–18 years old) parent pairs with a last recorded child BMI >95^th^ percentile and child cholesterol >169 mg/dL were randomized to PB or AHA with weekly 2-hour classes of nutrition education.

**Results:**

Children on PB had nine and children on AHA had four statistically significant (P<0.05) beneficial changes from baseline (mean decreases): BMI Z-score^PB^ (−0.14), systolic blood pressure^PB^ (−6.43 mm Hg), total cholesterol^PB^ (−22.5 mg/dL), low density lipoprotein^PB^ (−13.14 mg/dL), hsCRP^PB^ (−2.09 mg/L), insulin^PB^ (−5.42uU/ml), myeloperoxidase^PB/AHA^ (−75.34/69.23 pmol/L), mid-arm circumference^PB/AHA^ (−2.02/−1.55 cm), weight^PB/AHA^ (−3.05/ −1.14kg) and waist circumference^AHA^ (−2.96 cm). Adults on PB and AHA had seven and two respectively statistically significant (P<0.05) beneficial changes. The significant change favoring AHA was a 1% difference in children’s waist circumference. Difficulty shopping for food for the PB was the only statistically significant acceptability barrier.

**Conclusions:**

PB and the AHA in both children and adults demonstrated potentially beneficial changes from baseline in risk factors for CVD. Future larger, long-term randomized trials with easily accessible PB foods will further define the role of the PB in preventing CVD.

There is a need to have effective lifestyle modifications that target the growing group of obese children with dyslipidemia. The beneficial health effects of plant-based diets in adults are known. Studies have suggested that a low-fat, vegan diet (no animal products) may promote weight loss, lower body mass index (BMI), and improve lipoprotein profiles and insulin sensitivity and possibly prevent CVD.^6–10^ Those who follow a vegetarian diet (no animal products except for dairy and/or eggs) typically have lower cholesterol levels and a lower risk for coronary heart disease than non-vegetarians.^11–13^ Additionally, vegetarian diets have been shown to not only prevent but reverse heart disease in adults.^15, 16^

Whether the benefits of a plant-based (only plants and whole grains, limited avocado and nuts) no added fat diet (PB) extend to children is not known. We therefore conducted a four-week randomized trial comparing a PB with the American Heart Association diet (AHA)^17^ in children ages 9–18 with BMI >95% and total cholesterol >169mg/dL and one of their parents. Similar to the PB, the AHA encourages fruits, vegetables, whole grains and low sodium intake but permits non-whole grains, low-fat dairy, selected plant oils, and lean meat and fish in moderation.

The aim of this study was to determine if a PB and/or AHA significantly change anthropometric measurements and/or biomarkers of inflammation and CVD risk after a 4-week intervention in obese, hypercholesterolemic children age 9–18 years old and one of their parents. Our hypothesis was that both groups would show improvement in the studied outcomes and the improvements might be greater for the PB than AHA.

## METHODS

This was a prospective randomized 4-week trial (from April 20, 2013, to May 18, 2013) of either a PB or AHA. It was approved by our institutional review board. We enrolled 30 children seen in a large Midwestern hospital system’s predominantly middle class pediatric practices between the ages of 9–18 years with a last recorded BMI greater than the 95^th^ percentile for age and sex and most recent total cholesterol greater than 169 mg/dL. A parent or guardian also participated in the study and was assigned to follow the same diet that was given to his/her child to help with dietary compliance. Pregnant women were excluded from the study.

**Figure 1:**
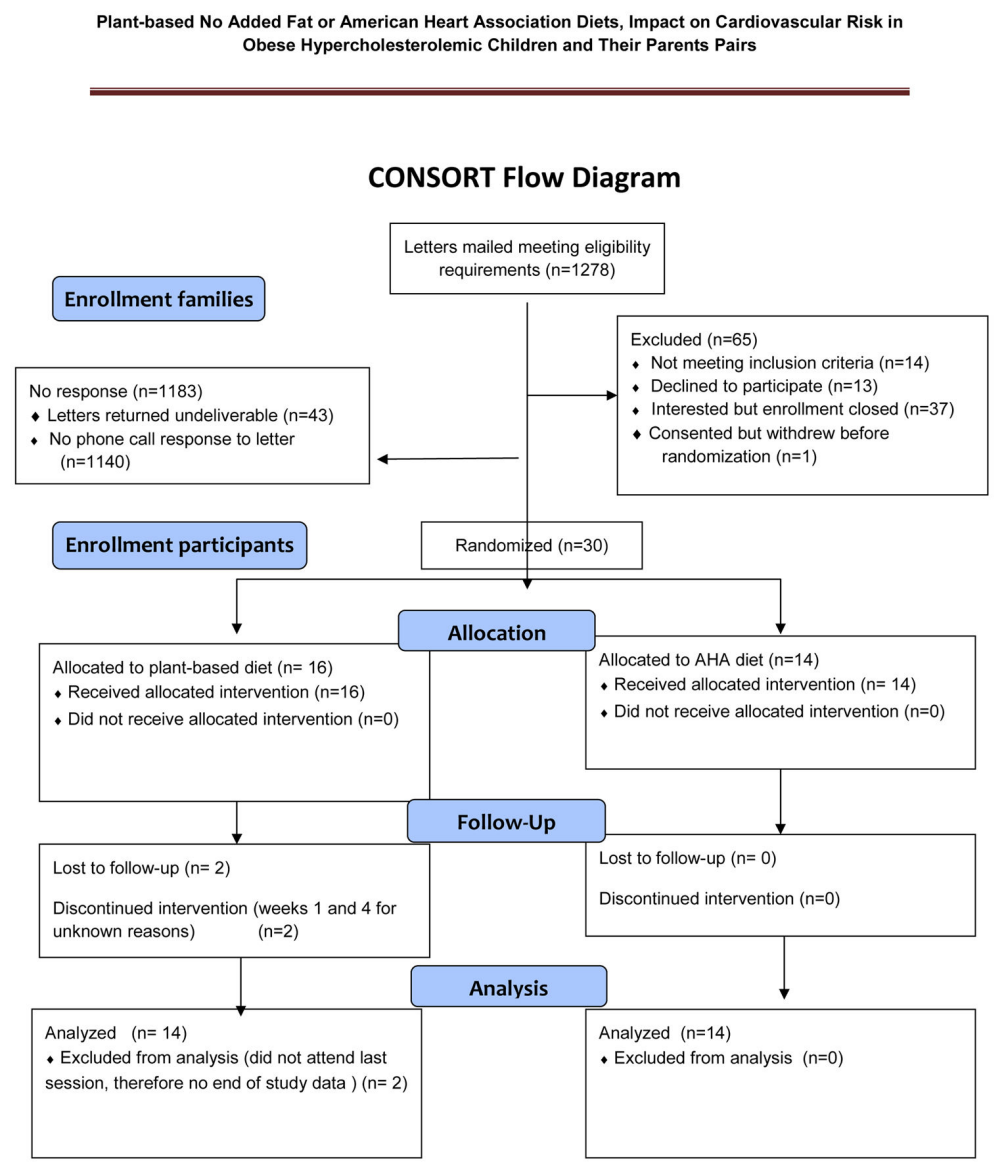
Consort Diagram

A computerized search of Cleveland Clinic medical records identified 1,278 potential participants (Figure 1; available at www.jpeds.com). Eligible patients were invited by letter to participate in the study. Those interested contacted the principal investigator (PI) and were enrolled on a first come, first served basis. Informed consent was obtained from the participants who were eighteen years of age and older. Participants younger than eighteen years provided assent with parent/guardian approval. There was a time gap between the last recorded measurements, obtaining informed consent, and the start of the study. During this time gap before the start of the study 6 previously obese (BMI >95%) children had become overweight (BMI 85%–95%), and one hypercholesterolemic (>169 mg/dL) child’s cholesterol had decreased to 169 mg/dL. Each child and parent pair received a fifty dollar stipend for each of the four weeks of the study.

Participants assigned to the PB were instructed to avoid all animal products and added fat, and to limit intake of nuts and avocado.^15^ The AHA group was allowed 30% of calories from total fat, 7% of calories from saturated fat, less than 300 mg cholesterol and less than 1,500 mg of sodium daily.^17^ All participants received standardized teaching at the time of consent to learn how to record a 24-hour dietary history. Participants completed two 3-day dietary histories consisting of two weekdays and one weekend day; one before the start of the study and one during the study. During the study, participants attended a total of 4 weekly 2-hour classes specific to their assigned diet consisting of one hour of nutrition education and one hour of cooking lessons with recipes provided.

Classes were led by acknowledged study collaborators. Weeks one and two focused on reading labels, where to buy food, food preparation, and how to stay on the assigned diets when eating away from home. Weeks three and four reviewed healthy food choices, the effects of diet on health, discussions of what worked and what did not work for the study participants. At the fifth and final study session, after all laboratory samples and measurements were obtained, participants had the option to attend an introductory class on the diet they were not assigned.

At the start of the 4-week trial, fasting blood samples for biomarkers of inflammation, and CVD risk were obtained. The biomarkers of inflammation were myeloperoxidase [MPO] and high sensitivity C-reactive protein (hsCRP)— which are biomarkers for inflammation and cardiovascular risk in prepubescent obese children and adults,^18, 19^ as well as IL-6, ALT, and AST. The CVD risk biomarkers were total cholesterol, triglycerides, high-density lipoprotein cholesterol (HDL-C), and low-density lipoprotein cholesterol (LDL-C), and included biomarkers for diabetes; HgbA1c, fasting glucose and insulin. Laboratory analysis included total cholesterol, triglycerides, low-density lipoprotein cholesterol (LDL-C), high-density lipoprotein cholesterol (HDL-C) by standard enzymatic methodology, hemoglobin A1c (HgbA1c) (in percentages %) by turbidimetric inhibition immunoassay, insulin by chemiluminescence immunoassay, high sensitivity C-reactive protein (hs-CRP) by immunoturbidometric assay and fasting plasma glucose by glucose hexokinase method.

All analyses were performed in the Preventive Research Laboratory and Lab Diagnostic Core, Cleveland Clinic.

Measurements (height, weight, mid-arm circumference, waist circumference, and blood pressure) were also obtained at the start of the trial. BMI was calculated by dividing weight in kilograms by height in meters squared. Measurements of the physical activity of the children and adolescents were self-reported using the Physical Activity Questionnaire.^20^ The PAQ consists of 9 questions which ask subjects to rate their physical activity for the previous 7 days, at different times of day and days of week, and how often they engaged in specific activities. All items are presented on a 5-point scale where 1 is low activity and 5 is high activity; the overall PAQ score is a mean of the 9 questions. All measurements were repeated at the completion of the study for comparison with baseline. Race/ethnicity was self-reported to help determine the comparability of the study groups. At the conclusion of the four-week trial, all participants completed a validated Food Acceptability Questionnaire,^21^ which subjectively rated the ease of following their assigned diets and their general like or dislike of the diet.

The sample size of 15 adults and children per group was calculated to substantially exceed, even with a 20% drop-out rate, the 6–7 patients per group required to provide a power of 90% at a significance level of 0.05 to detect the within-group changes from baseline in total cholesterol described previously (mean ± standard deviation decrease of 60±26 mg/dl)^14^ versus a null hypothesis mean decrease of 25±26mg/dl. We did not power our study to demonstrate statistically significant differences between two effective dietary interventions. Families were randomized to the two study groups in a 1:1 ratio in blocks of four families, stratified by the child’s age group (age strata 9–13 years vs. 14–18 years). The randomization was performed by Ms. Worley using an SAS computer program between the end of enrollment and the first weekly session.

Demographics, comorbidities, and body measurements were collected in a REDCap database,^22^ using double data entry. Laboratory values were provided in an Excel sheet. Diet journals were entered into and analyzed using Nutrition Data System for Research (NDSR) software.

### STATISTICAL ANALYSES

Mean daily nutrients were computed for each subject within the pre-study and during-study periods. The BMI of the children was converted to age- and sex- adjusted percentiles and their corresponding z-scores; statistical analysis was performed on the z- scores. Parent and child subjects were analyzed separately because their outcomes were likely to be correlated, given genetic and environmental similarities. For the primary analysis, within- group changes from baseline to week 4 were computed and their means estimated with 95% confidence intervals; log-transformations of baseline and week 4 values of variables were performed as needed. For the secondary analysis, the PB and AHA groups were compared at the end of the trial, adjusting for baseline values, using analysis of covariance (ANCOVA) models. Where needed to meet model assumptions, both the baseline and week 4 values of variables were log-transformed. Study groups were compared on responses to each question on the food acceptability questionnaire using Fisher’s exact tests and Cochran-Armitage trend tests. Sample sizes for individual variables reflect missing data. All analyses were performed on a complete- case basis. All tests were two-tailed and performed at a significance level of 0.05. SAS 9.2 software (SAS Institute, Cary, NC) was used for all analyses and R 3.0.0 (The R Foundation for Statistical Computing) was used for plots.

## RESULTS

Sixteen families were randomized to the PB and 14 families to the AHA. Two families, both in the PB group were lost to follow-up. One discontinued after the first week, and the other after the third week. Both families were excluded from the analysis because no end of study data was available. The final study cohort consisted of 28 families, 14 in each group (Figure 1). There were no significant between group differences in baseline demographic, nutrient, and clinical outcomes (Tables I–III; Tables II and III available at www.jpeds.com).

**Figure 2:**
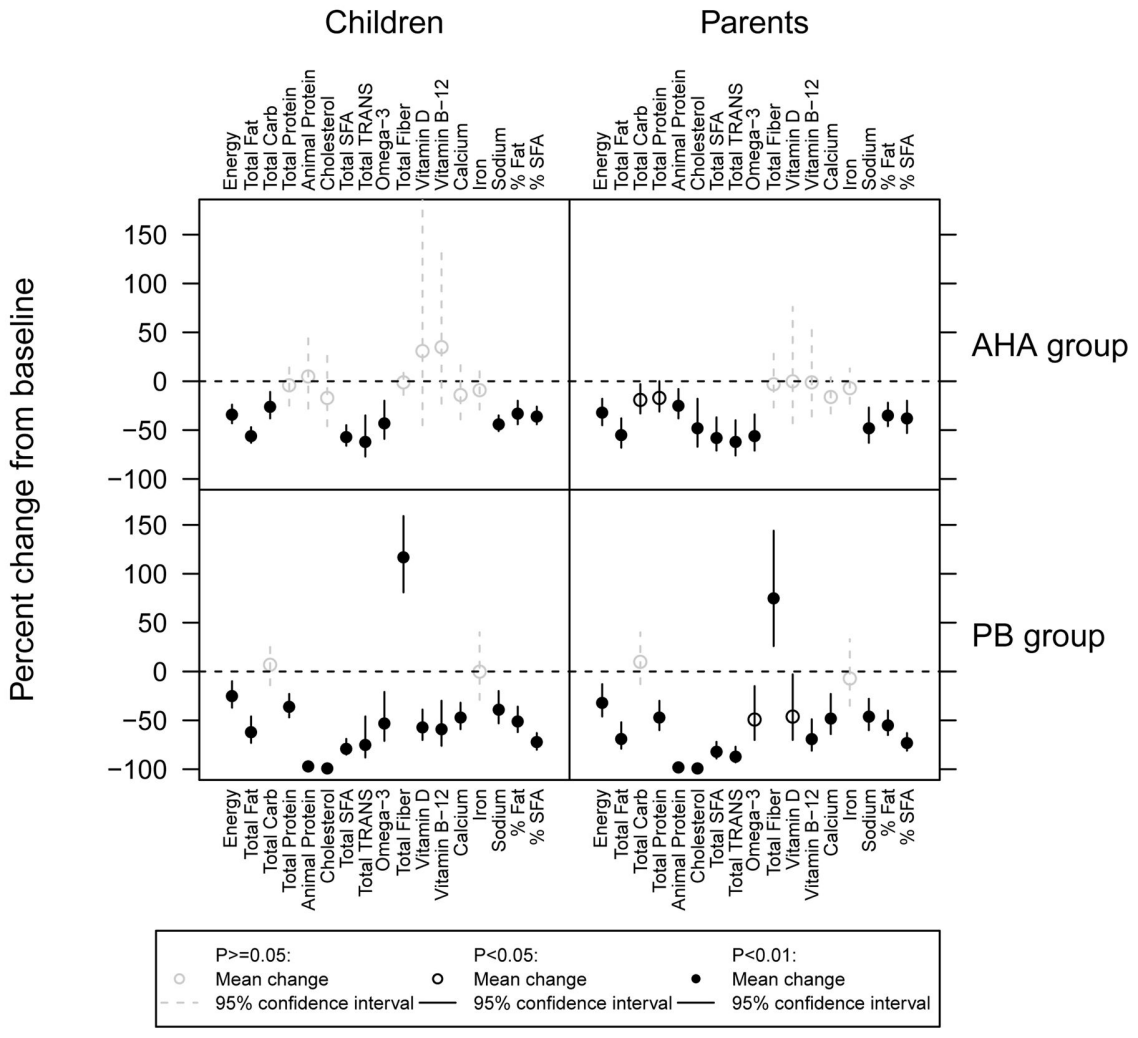
Nutrient Outcomes Within Groups SFA-Saturated Fatty Acids, Trans-Trans Fats

The total energy intake and the intake of almost all measured nutrients significantly decreased in children and adults in both groups, and dietary fiber intake significantly increased only in PB diet group (both children and adults) based on dietary histories completed during the study compared with those completed at baseline (Figure 2 and Table II). When comparing the PB and AHA groups during the study, children and adults in the PB group had a significantly lower intake of total protein, animal protein, cholesterol, total saturated fat, vitamin D, vitamin B12, percent of calories from fat and percent of calories from saturated fat. Children and adults in the PB group also had a significantly higher intake of total carbohydrates and dietary fiber than children in the AHA group. During the study, children and adults of both groups significantly reduced and increased intakes of the same nutrients, except for a trans-fat decrease only in adults on PB. Within-study energy intake and total fat intake were not significantly different between the two study groups, in children or adults.

Two goals for the children on PB were to consume no animal products and add no fat. During the study the mean (standard deviation) daily reported animal protein intake decreased from 42.32 (13.21) g to 2.24 (4.45) g (P <0.001) and the % of calories from fat and saturated fat was 18.04% (8.56%) and 3.59% (2.17%) respectively. The goals for the AHA children’s group were to consume <30% of total calories from fat, <7% of calories from saturated fat, <1,500 mg sodium, and <300 mg cholesterol. The respective mean (standard deviation) reported values during the study were 25.38% (6.12%), 7.59% (2.38%), 1,699 (897.71) mg and 144 (105.57) mg. Adults in both groups reported similar changes (Table II). These results suggest good but not perfect compliance with the assigned diets.

On The Food Acceptability Questionnaire^18^ using a seven-point response scale both children and parents in the PB group reported more difficulty purchasing the necessary food for their diet than the children and parents in the AHA group. Median difficulty ratings were 3 (“Slightly difficult”) in the PB group and 5 (“Fairly easy”) in the AHA group for both parent and child subject. Mean PB vs. AHA ratings were 3.7 vs. 5.1 for children and 3.5 vs. 5.1 for parents. There were no other statistically significant differences between the groups on how well they liked these foods, liked the taste, appearance appeal, how boring, ease of preparation, ease of maintaining diet at restaurants, effort to stay on diet, effect on cost of food purchases, satisfaction felt after meals, and overall satisfaction.

**Figure 3:**
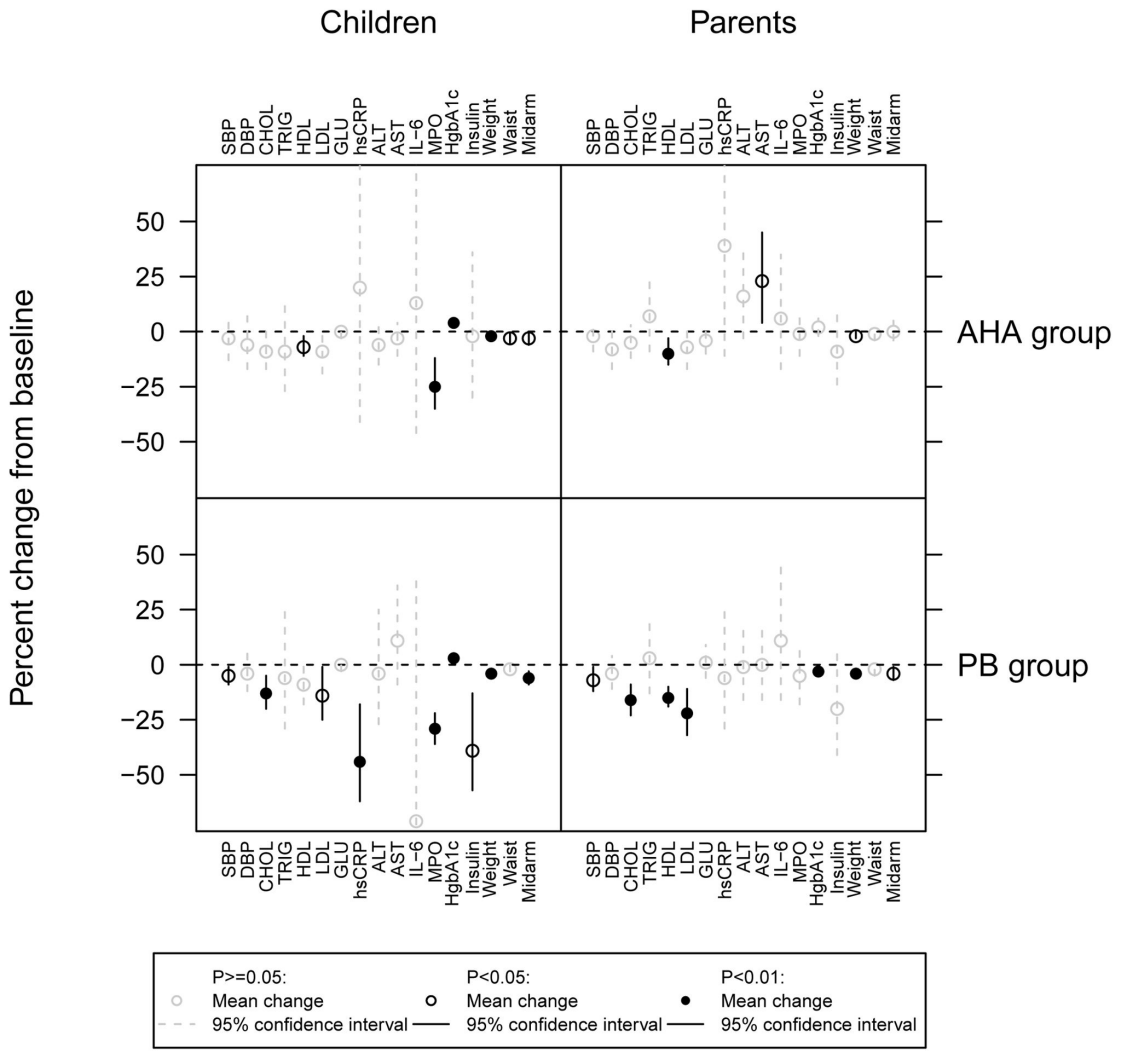
Clinical Outcomes Within Groups SBP-Systolic Blood Pressure, DBP-Diastolic Blood Pressure

In the group of children on PB, there were statistically significant (P<0.05) mean decreases in nine measures: BMI Z-score (−0.14), systolic blood pressure (−6.43 mm Hg), weight (−3.05 kg), mid-arm circumference (−2.02 cm), total cholesterol (−22.5 mg/dL), LDL (−13.14 mg/dL), hsCRP (−2.09 mg/L), MPO (−75.34 pmol/L), and insulin (−5.42 *u*U/ml). In the AHA children group, there were statistically significant mean decreases in 5 measures: weight (−1.55 kg), waist circumference (−2.96 cm), mid-arm circumference (−1.14 cm), high density lipoprotein (−2.93 mg/dL), and MPO (−69.23 pmol/L). Both groups had statistically significant increases in Hgb A1c (PB +0.17%, AHA +0.21%) (Figure 3 and Table III). In the group of adults on PB, there were statistically significant (P<0.05) mean decreases in eight measures: BMI (−1.29kg/m^2^), systolic blood pressure (−7.96mm Hg), weight (−3.64kg), mid-arm circumference (−1.32cm), total cholesterol (−33.79mg/dL), HDL (−8.14mg/dL), LDL (−27.0mg/dL), and Hgb A1C (−0.16%). In the AHA adult group, there were statistically significant mean decreases in three measures: BMI (−0.73kg/m^2^), weight (−2.01kg), and HDL (−4.93mg/dL). The only statistically significant mean increase was AST (+4.43 U/L) in the AHA group (Figure 3 and Table III).

The only statistically significant differences between the PB and AHA groups after the intervention were that the children in the PB group had significantly lower week 4 BMI Z-scores and hsCRP levels. Parents in the PB group had significantly lower total cholesterol, LDL, and Hgb A1C than parents in the AHA group. The only significant change favoring AHA was a 1% difference in children’s waist circumference.

The primary analysis of our study was of evaluable cases.

In this study of PB in children we were most interested in determining if adhering to a PB would improve cardiovascular risk. A secondary intent-to-treat analysis was performed including the two child/parent pairs on PB who failed to complete the study. For those pairs, we assumed that there was no change from baseline in outcomes where baseline measures were available, and for measures with unavailable baseline data (insulin, IL-6, and myeloperoxidase), we assigned the median baseline value of the measure, computed separately for all parent and all child subjects, to both the baseline and end of study values. There were no differences between the evaluable-case analysis and intent-to-treat analysis in the statistical significance of within- group changes in outcome. The only differences between the evaluable-case analysis and intent- to-treat analysis in the statistical significance of the PB and AHA group comparisons were that children in the PB group no longer had significantly lower week 4 hsCRP or vitamin D intake, and parents in the PB no longer had significantly lower total protein or percent of calories from fat (data not shown).

## DISCUSSION

We believe our inability to demonstrate more than a few significant differences between our intervention groups, versus many significant differences from baseline values, most likely reflects the fact our study was powered to detect changes from baseline values in the PB group with 4 weeks intervention.^14^

Statistically significant differences that occurred between baseline and week 4 that might be of possible concern for increased cardiovascular risk include the elevation of Hgb A1c in both the plant-based and AHA children groups. However, the only statistically significant change in HOMA-IR in our study was in the PB children’s group with a mean ratio (95% CI) of −1.25 (−2.01, −0.99), P-value 0.004, which suggests significantly decreased insulin resistance. In the PB children’s group FIRI (fasting immunoreactive insulin) and in the adult PB group HgbA1c decreased significantly. All these values suggest, if anything, a decreased risk of diabetes in the PB group. Other studies have documented beneficial effects of PB on diabetes.^6, 7, 24^ The statistically significant decreases of HDL in both the adult and children AHA group and the adult PB group are also of some concern. The decrease in serum HDL cholesterol after 4 weeks was most likely associated with early weight loss.^25^ Vegan diets have been previously reported to be associated with decreased HDL, but vegan diets are also associated with a decreased risk of heart disease.^8,15, 26^ However, we are unaware of similar reports of lowering HDL on the AHA diet. There is heterogeneity described in the composition and function of HDL, and further characterization of HDL after exposure to these diets, might prove helpful in establishing the significance of these findings of decreased HDL.^27, 28^ The statistically significant elevation of AST in the adults on the AHA diet is of unknown significance. Mild, transient increases in ALT and AST values have been observed in weight loss studies, but usually returned to below baseline levels after substantial weight loss.^29, 30^

Statistically significant reported differences in the PB and AHA diet in adults and children of decreased cholesterol, fat and calorie intake and increased fiber on the PB diet most likely were of benefit to the obese hypercholesterolemic children studied.^9, 24^ Previous reports demonstrate that dietary records, especially in individuals with higher BMI, commonly underestimate intake by nearly 50%, so the actual intake is difficult to know for certain. The reported decrease of protein intake, especially in the context of probable under-reporting of intake, is not of concern as PB diets have been shown to provide adequate protein and most other nutrients in adults and children.^31–33^ The decreased intake of Vitamins B12 and D reported in the adult and child PB groups found in the current study have been noted previously. Vitamin B 12 definitely and Vitamin D probably warrant supplements for those on long-term PB diets. Intake of key nutrients is generally adequate in a balanced vegan diet, but it is still essential to monitor consumption of protein, n-3 fatty acids, iron, zinc, iodine, and calcium in long-term vegans.^31–33^ Dietary intake of n-3 long-chain polyunsaturated fatty acids (PUFAs) eicosapentaenoic acid and docosahexaenoic acid, is low in vegans compared with omnivores.^34^ Therefore, n-3 fatty acids, particularly in a no added fat vegan diet, should be especially carefully monitored and may also require supplementation with algae-derived n-3 PUFAs. The only significant described problem in our middle class study population for diet acceptance was the difficulty purchasing food. Cost may be a barrier to a PB in lower socioeconomic populations. In another study the only identified barrier to adherence was the effort required.^35^ If the PB diet is to achieve ever increasing adaptation, barriers to easy, affordable access to plant-based, no added fat foods will need to be reduced.

The major limitations of our study are that this was a small study conducted for a short period of time in a select group of middle class patients with less than completely reliable measures of compliance and with no direct health outcomes measures. Also, the AHA is considered a standard of care and was used as a comparison group—there was no placebo- controlled group. There is also concern that long-term compliance with the PB could be problematic. This is especially true given the difficulties expressed by parents and children in finding food to purchase for the diet in our study, and in the effort required to maintain a PB in a previous study.^21^ However, there are studies describing good acceptability and compliance with a PB. ^15,16,35,36^

Plant-based diets are generally recognized as safe for children and adolescents as long as the intake of key nutrients is monitored and appropriate supplements are provided. The results of our study suggest that the documented benefits of PB in adults, including, but not limited to, decreased overweight and obesity and decreased cardiovascular risk, most likely would be seen in children. These benefits, especially given the known onset of CVD in childhood, could improve the lifetime health of those populations who choose to eat a PB beginning in childhood.

## Figures and Tables

**Table 1:** Demographics

Factor	Plant-based (N=14)	AHA (N=14)
**Child: male**	5(36)	5(36)
**Child: Age, years**	15.0(9.0,18.0)	15.0(9.0,18.0)
**Child: race**		
• **None given**	1(7)	0(0)
• **White**	10(71)	10(71)
• **Black**	2(14)	4(29)
• **Asian**	1(7)	0(0)
Child: Hispanic ethnicity*	0(0)	1(9)
**Child: BMI at baseline**		
• **Overweight**	4(29)	2(14)
• **Obese**	10(71)	12(86)
**Child: Blood pressure at baseline**		
• **Normal**	8(57)	8(57)
• **Prehypertension**	5(36)	4(29)
• **Hypertension**	1(7)	2(14)
**Child: High cholesterol at baseline (>169)**	14(100)	13(93)
**Parent: male**	5(36)	4(29)
**Parent: Age (years)**	46.5(37.0,61.0)	46.0(35.0,51.0)
**Parent: race**		
• **White**	10(71)	9(64)
• **Black**	3(21)	4(29)
• **Asian**	1(7)	0(0)
• **More than one race**	0(0)	1(7)
Parent: Hispanic ethnicity*	0(0)	1(10)
**Parent: BMI at baseline**		
• **Normal weight**	1(7)	3(21)
• **Overweight**	4(29)	3(21)
• **Obese**	9(64)	8(57)
**Parent: Blood pressure at baseline**		
• **Normal**	7(50)	3(21)
• **At risk**	3(21)	8(57)
• **High**	4(29)	3(21)
**Parent: High cholesterol at baseline (>199)**	8(57)	8(57)

* Data not available for all subjects. Missing values: Child: Hispanic ethnicity = 6, Parent: Hispanic ethnicity = 8.

Values presented as Means with SDs indicated in parenthesis, Median [P25, P75], Median (min, max) or N (column %).

**Table 2:** Nutrients Compared Between Plant-based and American Heart Association Diets

CHILDREN									
Energy (kcal)	1686.32 (650.59)	1208.02 (288.96)	−478.30 (496.93)	1638.16 (484.17)	1115.90 (462.71)	−522.26 (289.68)		1.14 (0.93, 1.38)	0.19
Outcome	Plant-Based (N=14) Mean (SD)			AHA (N=14) Mean (SD)			PB - AHA Adjusted Mean Difference (95% CI) at Week 4	PB/AHA Adjusted Mean Ratio (95% CI) at Week 4	P- value
	Baseline	During study	Change	Baseline	During study	Change			
Total Fat (g)	65.72 (30.97)	26.60 (17.90)	−39.12 (26.57)	69.66 (20.34)	33.39 (18.31)	−36.27 (12.93)		0.82 (0.57, 1.19)	0.29
Total Carbohydrate (g)	217.35 (97.86)	215.83 (58.11)	−1.52 (74.58)	198.09 (73.71)	152.39 (73.06)	−45.70 (46.51)	53.18 (15.48, 90.88)		0.008
Total Protein (g)	63.61 (17.76)	39.61 (6.92)	−24.00 (16.98)	60.06 (20.81)	56.38 (17.83)	−3.68 (20.89)	−17.69 (−27.70, −7.68)		0.001
Animal Protein (g)	42.32 (13.21)	2.24 (4.45)	−40.08 (14.35)	38.61 (17.38)	38.25 (15.48)	−0.36 (21.99)	−36.19 (−45.28, −27.10)		<0.001
Cholesterol (mg)	181.23 (81.81)	12.93 (30.63)	−168.30 (79.95)	169.90 (84.68)	143.70 (105.57)	−26.20 (104.27)	−134.32 (−192.59, −76.05)		<0.001
Total Saturated Fatty Acids (SFA) (g)	21.97 (8.78)	5.46 (4.52)	−16.51 (8.51)	22.41 (11.32)	10.20 (6.75)	−12.22 (7.32)		0.49 (0.33, 0.75)	0.002
Total Trans-Fatty Acids (TRANS) (g)	2.11 (1.22)	0.80 (1.03)	−1.31 (1.63)	2.69 (1.24)	1.05 (0.54)	−1.64 (1.28)		0.51 (0.24, 1.08)	0.078
Omega-3 Fatty Acids (g)	1.33 (1.16)	0.62 (0.51)	−0.71 (1.17)	1.57 (0.83)	0.87 (0.41)	−0.70 (0.81)	−0.22 (−0.58, 0.13)		0.21
Total Dietary Fiber (g)	13.68 (4.27)	29.01 (7.91)	15.32 (6.30)	12.92 (5.15)	13.58 (7.65)	0.66 (3.71)		2.22 (1.79, 2.75)	<0.001
Vitamin D (calciferol) (mcg)	4.08 (1.88)	1.47 (1.44)	−2.61 (1.65)	3.78 (4.21)	2.45 (1.66)	−1.33 (4.38)		0.53 (0.31, 0.92)	0.026
Vitamin B-12 (cobalamin) (mcg)	4.75 (3.47)	2.03 (2.55)	−2.72 (3.33)	3.08 (1.69)	2.96 (1.40)	−0.12 (1.74)		0.45 (0.25, 0.82)	0.011
Calcium (mg)	806.89 (327.11)	395.80 (84.63)	−411.09 (297.61)	611.89 (315.44)	493.82 (249.68)	−118.07 (309.23)		0.78 (0.60, 1.02)	0.069
Iron (mg)	16.32 (10.20)	15.39 (6.58)	−0.92 (11.38)	11.72 (4.88)	10.33 (5.13)	−1.39 (3.97)	4.12 (−0.57, 8.82)		0.082
Sodium (mg)	2841.92 (962.40)	1697.75 (401.15)	−1144.17 (1160.52)	2842.88 (886.84)	1699.27 (897.71)	−1143.61 (473.64)	−1.20 (−499.33, 496.93)		0.99
% Calories from Fat	33.48 (6.80)	18.04 (8.56)	−15.44 (8.24)	36.99 (6.67)	25.38 (6.12)	−11.61 (8.50)	−6.15 (−11.98, −0.32)		0.039
% Calories from SFA	11.78 (4.15)	3.59 (2.17)	−8.19 (4.39)	11.36 (2.47)	7.59 (2.38)	−3.77 (1.80)		0.43 (0.31, 0.59)	<0.001
**PARENTS**									
Energy (kcal)	1964.39 (950.71)	1235.70 (335.76)	−728.70 (839.79)	1660.18 (422.06)	1142.41 (368.92)	−517.77 (460.19)		1.07 (0.86, 1.32)	0.54
Total Fat (g)	84.93 (50.55)	25.84 (14.22)	−59.09 (50.54)	70.90 (26.34)	33.93 (20.18)	−36.96 (29.81)		0.75 (0.49, 1.13)	0.16
Total Carbohydrate (g)	218.33 (101.44)	222.89 (67.31)	4.56 (81.29)	190.82 (47.77)	158.45 (54.82)	−32.38 (48.99)	52.30 (11.62, 92.97)		0.014
Total Protein (g)	83.12 (36.04)	41.33 (11.46)	−41.79 (34.43)	69.31 (18.91)	57.32 (13.69)	−11.99 (19.44)	−17.71 (−27.61, −7.80)		0.001
Animal Protein (g)	54.22 (26.26)	1.30 (2.27)	−52.93 (25.89)	46.43 (16.55)	35.40 (13.93)	−11.03 (12.93)	−35.41 (−42.90, −27.93)		<0.001
Cholesterol (mg)	241.61 (161.29)	7.93 (14.13)	−233.69 (155.19)	212.75 (89.96)	119.64 (67.33)	−93.11 (117.02)	−112.16 (−151.00, −73.33)		<0.001
Total Saturated Fatty Acids (SFA) (g)	28.14 (17.45)	5.03 (4.21)	−23.11 (16.49)	22.60 (9.85)	10.12 (5.62)	−12.48 (11.48)		0.45 (0.29, 0.72)	0.002
Total Trans-Fatty Acids (TRANS) (g)	3.15 (2.21)	0.47 (0.28)	−2.68 (2.24)	3.07 (2.57)	1.04 (0.49)	−2.03 (2.62)		0.35 (0.18, 0.69)	0.004
Omega-3 Fatty Acids (g)	1.62 (0.62)	0.94 (0.67)	−0.69 (0.96)	1.63 (0.68)	0.84 (0.72)	−0.79 (0.69)	0.10 (−0.44, 0.64)		0.71
Total Dietary Fiber (g)	20.11 (9.59)	31.99 (8.24)	11.88 (11.32)	17.52 (7.53)	16.42 (6.50)	−1.10 (7.22)		1.99 (1.55, 2.55)	<0.001
Vitamin D (calciferol) (mcg)	2.67 (1.82)	0.96 (1.39)	−1.72 (2.35)	3.71 (3.32)	2.87 (2.64)	−0.84 (3.98)		0.45 (0.25, 0.80)	0.008
Vitamin B-12 (cobalamin) (mcg)	3.97 (2.35)	1.01 (1.72)	−2.97 (2.54)	3.37 (1.56)	3.38 (3.04)	0.02 (3.35)		0.32 (0.18, 0.58)	<0.001
Calcium (mg)	780.10 (333.35)	387.65 (170.62)	−392.45 (350.28)	572.29 (123.52)	524.45 (245.01)	−47.84 (160.68)		0.71 (0.49, 1.01)	0.059
Iron (mg)	14.76 (6.30)	14.94 (13.51)	0.19 (13.37)	11.54 (3.01)	11.19 (4.12)	−0.35 (3.21)		1.10 (0.77, 1.57)	0.58
Sodium (mg)	3515.11 (1899.46)	1708.01 (464.17)	−1807.11 (1748.47)	3235.59 (1109.71)	1754.93 (787.72)	−1480.66 (1241.75)	−78.00 (−574.67, 418.67)		0.75
% Calories from Fat	35.73 (7.37)	17.40 (6.81)	−18.33 (7.69)	36.58 (6.90)	24.44 (7.32)	−12.14 (8.71)	−6.77 (−12.07, −1.46)		0.015
% Calories from SFA	11.69 (4.22)	3.31 (1.73)	−8.38 (3.78)	11.66 (2.91)	7.30 (2.16)	−4.36 (4.01)	−3.99 (−5.53, −2.45)		<0.001

**Table 3:** Clinical Outcomes

Outcome	Plant-Based (N=14) Mean (SD)			AHA (N=14) Mean (SD)			PB - AHA Adjusted Mean Difference (95% CI) at Week 4	PB/AHA Adjusted Mean Ratio (95% CI) at Week 4	P-value
	Baseline	Week 4	Change	Baseline	Week 4	Change			
**CHILDREN**									
BMI percentile	96.36 (2.63)	95.24 (2.89)	−1.12 (1.69)	98.01 (1.81)	97.93 (1.79)	−0.08 (0.42)	−1.17 (−2.20, −0.14)		0.028
BMI Z score	1.90 (0.38)	1.77 (0.39)	−0.14 (0.16)	2.19 (0.38)	2.17 (0.37)	−0.03 (0.07)	−0.13 (−0.24, −0.03)		0.017
Systolic BP (mm Hg)	116.25 (14.10)	109.82 (9.67)	−6.43 (8.86)	111.50 (20.16)	106.36 (9.87)	−5.14 (17.64)	1.87 (−4.41, 8.15)		0.55
Diastolic BP (mm Hg)	68.79 (9.61)	66.18 (8.75)	−2.61 (10.25)	69.79 (11.56)	65.43 (7.43)	−4.36 (14.67)		1.01 (0.92, 1.11)	0.83
Weight (kg)	79.62 (23.43)	76.57 (23.38)	−3.05 (3.27)	87.71 (19.85)	86.16 (19.01)	−1.55 (1.83)	−1.71 (−3.80, 0.39)		0.11
Waist circ (cm)	97.11 (12.05)	95.58 (12.22)	−1.53 (3.01)	104.89 (11.99)	101.93 (12.53)	−2.96 (4.04)	1.32 (−1.66, 4.30)		0.37
Midarm circ (cm)	33.30 (5.02)	31.29 (4.25)	−2.02 (2.17)	35.06 (3.64)	33.91 (3.37)	−1.14 (1.63)	−1.25 (−2.61, 0.11)		0.070
PAQ	2.44 (0.63)	2.66 (0.83)	0.22 (0.53)	2.36 (0.74)	2.20 (0.63)	−0.16 (0.54)	0.39 (−0.02, 0.80)		0.060
CHOL (mg/dL)	213.79 (41.97)	191.29 (70.34)	−22.50 (36.31)	194.07 (25.69)	177.57 (25.53)	−16.50 (28.19)	−10.34 (−36.48, 15.80)		0.42
TRIG (mg/dL)	139.93 (139.03)	114.43 (89.77)	−25.50 (95.26)	119.29 (63.31)	106.14 (52.58)	−13.14 (36.19)		1.01 (0.77, 1.32)	0.94
HDL (mg/dL)	55.43 (19.10)	49.50 (16.11)	−5.93 (9.37)	43.36 (6.96)	40.43 (6.17)	−2.93 (3.71)	0.17 (−5.07, 5.40)		0.95
LDL (mg/dL)	132.21 (29.04)	119.07 (54.22)	−13.14 (35.54)	126.86 (19.06)	115.86 (18.45)	−11.00 (23.31)		0.95 (0.80, 1.13)	0.54
GLU (mg/dL)	98.86 (45.38)	99.79 (48.89)	0.93 (6.01)	98.57 (49.50)	97.93 (46.81)	−0.64 (5.02)		1.01 (0.96, 1.05)	0.81
hsCRP (mg/L)	5.11 (8.33)	3.03 (4.64)	−2.09 (7.10)	4.09 (3.76)	6.87 (8.63)	2.78 (8.76)		0.46 (0.21, 1.00)	0.049
ALT (U/L)	18.50 (6.64)	19.29 (14.14)	0.79 (14.35)	25.36 (27.45)	24.21 (28.33)	−1.14 (3.46)		1.00 (0.76, 1.32)	0.98
AST (U/L)	18.43 (3.55)	21.21 (8.40)	2.79 (8.96)	22.50 (13.95)	22.50 (17.77)	0.00 (4.69)		1.13 (0.92, 1.40)	0.23
IL-6 (pg/ml)	1.72 (1.10)	1.55 (1.34)	−0.17 (0.89)	2.33 (2.50)	2.14 (1.97)	−0.19 (3.14)		0.26 (0.05, 1.37)	0.11
MPO (pmol/L)	287.54 (147.35)	212.20 (138.87)	−75.34 (37.34)	303.79 (89.92)	234.56 (87.95)	−69.23 (72.92)		0.95 (0.79, 1.13)	0.52
HgbA1c	5.77 (1.60)	5.94 (1.63)	0.17 (0.17)	6.14 (1.82)	6.34 (1.82)	0.21 (0.12)		0.99 (0.97, 1.01)	0.51
Insulin (uU/ml)	17.33 (14.12)	11.91 (9.40)	−5.42 (5.68)	23.19 (12.57)	26.36 (26.25)	3.16 (25.62)		0.70 (0.43, 1.12)	0.13
**PARENTS**									
BMI	33.26 (7.69)	31.98 (7.57)	−1.29 (1.14)	37.08 (12.66)	36.35 (12.20)	−0.73 (0.92)	−0.69 (−1.47, 0.09)		0.082
Systolic BP (mm Hg)	125.18 (16.98)	117.21 (17.18)	−7.96 (12.28)	121.64 (15.07)	118.50 (11.90)	−3.14 (14.42)		0.97 (0.89, 1.05)	0.39
Diastolic BP (mm Hg)	78.25 (12.06)	74.79 (8.75)	−3.46 (11.06)	79.29 (10.34)	72.64 (8.58)	−6.64 (12.19)		1.03 (0.94, 1.13)	0.46
Weight (kg)	93.33 (27.18)	89.70 (26.28)	−3.64 (3.41)	100.79 (32.16)	98.77 (30.73)	−2.01 (2.66)	−1.95 (−4.16, 0.26)		0.081
Waist circ (cm)	104.41 (15.05)	102.47 (18.20)	−1.94 (5.80)	112.11 (24.55)	111.62 (25.13)	−0.49 (5.73)	−1.14 (−5.75, 3.48)		0.62
Midarm circ (cm)	35.52 (6.61)	34.20 (6.19)	−1.32 (2.06)	35.89 (7.67)	36.24 (8.61)	0.35 (3.16)	−1.68 (−3.80, 0.44)		0.11
CHOL (mg/dL)	210.43 (51.63)	176.64 (40.80)	−33.79 (30.84)	214.43 (45.28)	207.29 (55.66)	−7.14 (24.93)	−27.29 (−48.68, −5.90)		0.014
TRIG (mg/dL)	130.07 (80.18)	136.29 (89.70)	6.21 (41.71)	117.21 (53.57)	134.07 (75.74)	16.86 (36.86)		0.95 (0.76, 1.19)	0.67
HDL (mg/dL)	56.14 (17.24)	48.00 (15.60)	−8.14 (5.40)	59.57 (13.47)	54.64 (15.70)	−4.93 (5.61)		0.94 (0.87, 1.02)	0.16
LDL (mg/dL)	128.36 (42.00)	101.36 (36.06)	−27.00 (26.72)	131.36 (43.67)	125.86 (50.02)	−5.50 (20.89)	−21.92 (−40.37, −3.46)		0.022
GLU (mg/dL)	101.50 (31.28)	106.43 (53.88)	4.93 (24.65)	100.64 (22.69)	95.21 (14.36)	−5.43 (12.40)		1.06 (0.97, 1.16)	0.20
hsCRP (mg/L)	3.60 (4.16)	3.36 (3.52)	−0.24 (1.36)	5.23 (6.20)	5.44 (5.15)	0.21 (1.90)		0.68 (0.44, 1.07)	0.091
ALT (U/L)	21.14 (8.73)	22.00 (13.69)	0.86 (7.01)	20.64 (7.43)	25.21 (13.73)	4.57 (9.69)		0.85 (0.67, 1.08)	0.17
AST (U/L)	18.93 (4.41)	19.07 (5.59)	0.14 (5.70)	18.14 (5.04)	22.57 (7.71)	4.43 (6.73)		0.83 (0.68, 1.03)	0.084
IL-6 (pg/ml)	7.86 (21.09)	8.02 (20.83)	0.16 (0.90)	2.13 (1.53)	1.94 (1.06)	−0.19 (0.85)		1.14 (0.83, 1.57)	0.40
MPO (pmol/L)	297.59 (145.78)	314.50 (236.87)	16.91 (110.66)	256.14 (70.52)	257.91 (87.62)	1.78 (45.93)		0.93 (0.80, 1.10)	0.39
HgbA1c	5.89 (0.97)	5.73 (0.88)	−0.16 (0.16)	5.81 (0.51)	5.94 (0.79)	0.14 (0.49)		0.96 (0.92, 1.00)	0.031
Insulin (uU/ml)	13.46 (8.25)	10.34 (6.67)	−3.11 (8.56)	16.33 (12.33)	13.18 (7.96)	−3.15 (5.47)		0.87 (0.67, 1.13)	0.27

## LIST OF ABBREVIATIONS AND ACRONYMS

CVD: Cardiovascular disease
PB: Plant-based (only plants and whole grains, limited avocado and nuts) no added fat diet
AHA: American Heart Association Diet (also encourages fruits, vegetables, whole grains and low sodium intake but permits non-whole grains, low-fat dairy, selected plant oils, and lean meat and fish in moderation.)
HOMA-IR: Homeostasis Model Assessment –Insulin Resistance=FIRI (fasting immunoreactive insulin mU/l)×FPG (fasting plasma glucose mmol/l)/22.5
